# A New Scoring System for Spontaneous Closure Prediction of Perimembranous Ventricular Septal Defects in Children

**DOI:** 10.1371/journal.pone.0113822

**Published:** 2014-12-05

**Authors:** Jing Sun, Kun Sun, Sun Chen, Liping Yao, Yuqi Zhang

**Affiliations:** 1 Pediatric Heart Center, Xinhua Hospital, School of Medicine, Shanghai Jiaotong University, Shanghai 200092, China; 2 Department of Echocardiography, Xinhua Hospital, School of Medicine, Shanghai Jiaotong University, Shanghai 200092, China; 3 Department of Pediatric Cardiology, Shanghai Children's Medical Center, School of Medicine, Shanghai Jiaotong University, Shanghai 200127, China; Bambino Gesù Children's Hospital, Italy

## Abstract

**Background:**

Perimembranous ventricular septal defect (PMVSD) is a congenital heart aberration, which is surgically treated by patch or device closure, but also can heal without operation as spontaneous closure (SC).

**Methods:**

We analyzed data from 1873 PMVSD patients admitted to our hospital during September 2001 and December 2009, in order to establish a Cox regression model for PMVSD SC probability prediction (derivative cohort). Initial contact age, ventricular septal defect (VSD) diameter, shunt flow, aneurysmal tissue of the ventricular membranous septum (ATVMS) development, associated complications, and left ventricular end-diastolic dimension (LVDD) were analyzed for correlations with SC. The derived scoring system based on the coefficients of the model was developed and applied to another cohort with 382 PMVSD patients to evaluate the validity for SC probability forecast (validation cohort).

**Results:**

Multivariate Cox regression analysis revealed that SC of PMVSD was associated with age at first contact, defect size, diffuse shunt flow, ATVMS formation, associated complication, as well as increased LVDD, which were used to establish a new scoring system. The area under the receiver operating characteristic (ROC) curve of our predictive scaling was 0.831 (95% CI 0.804–0.858, p<0.001) in the derivative cohort. The scoring system also accurately predicted SC with an area under the ROC curve of 0.863 (95% CI 0.785–0.941, p<0.001) in the validation cohort.

**Conclusion:**

Our scoring system using factors affecting SC can predict the probability of SC in PMVSD patients.

## Introduction

Ventricular septal defect (VSD) is the most frequently diagnosed congenital heart defect, with a reported overall incidence of 17.3 cases per 1000 births in China [1]. Depending on the follow-up period and type of perimembranous ventricular septal defect (PMVSD), the rate of spontaneous closure (SC) varies between 6% and 50% [2]–[7]. Although defect size [6], defect location [7], the presence of aneurysmal tissue of the ventricular membranous septum (ATVMS) [4] and associated complications [2], [4], [8] have been identified as factors for predicting spontaneous PMVSD closure, there is no scoring system that takes all of these factors with their various degrees into account for predicting the outcome of PMVSD. Previous models for forecasting SC probability and necessary VSD surgery were derived from indexed VSD cross-sectional area determinations and in a small group of patients [6]. In our study, we sought to develop a new scoring system for predicting spontaneous PMVSD probability and examine its validity.

## Patients and Methods

### Patients

**Figure 1:**
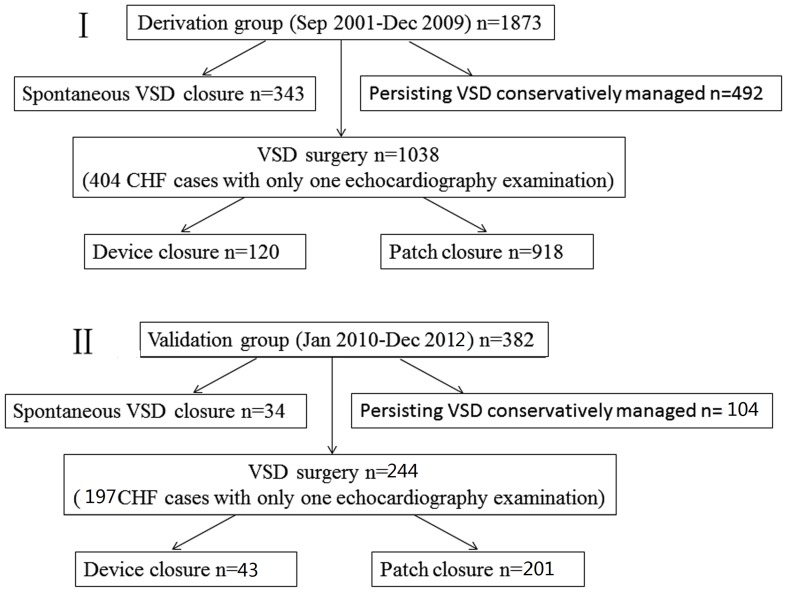
Flow chart of the two patient groups for establishment of a spontaneous PMVSD closure prediction scoring system. (VSD, ventricular septal defect; CHF, congestive heart failure).

The study was approved by the institutional review board of the Shanghai Jiaotong University and written informed consent was obtained from all parents. We included 2255 PMVSD patients who met the inclusion criteria in this study. Inclusion criteria were: 1) careful echocardiographic examinations; 2) integrated follow-up data; 3) presence of an isolated PMVSD determined by the clinical features and diagnostics evaluation [9] or PMVSD and additional heart abnormalities that did not obviously affect VSD hemodynamically [10], including 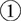 an open foramen ovale or a small secundum aerial septal defect, 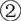 a small patent ductus arteriosus, and 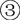 a persistent left superior vena cava. Exclusion criteria were multiple ventricular septal defects and complicated cardiac malformations, such as tetralogy of Fallot, pulmonary atresia, and truncus arteriosus. In the first part of our analysis, a derivative data set from 1873 PMVSD patients who first contacted Shanghai Xinhua Hospital between September 2001 and December 2009 was collected and used to develop a predictive scoring system for PMVSD SC (derivative patient group). In the second part of the study, the scoring system was then applied for 382 patients, who were admitted to the hospital between January and December 2010, in order to validate the derived scoring system (validation patient group, Figure 1).

### Echocardiography

Echocardiographic morphometric features recorded on videotape, as well as demographic data (sex, age at initial contact), were collected at the beginning of our study. Echocardiograms were performed using commercially available echocardiographic equipment with available transducers. Two dimensional, M-mode, and Doppler echocardiographic images were obtained at the subcostal, parasternal, apical, and suprasternal notch views. Defect diameters were measured as the mean interruption of ventricular septum of 2 orthogonal planes. Color Doppler flow mapping was also used to judge whether the shunt flow was diffuse. Left ventricular end-diastolic dimension (LVDD) was measured from M-mode tracings according to published guidelines [11], and then compared to the normal dimension of healthy children in China at the same age to judge whether it was increased [12]. A pulmonary artery systolic pressure (PASP)>36 mmHg or mean pulmonary artery pressure (PAPm)>25 mmHg at rest was diagnosed as pulmonary hypertension (PH) [13]. According to the different extensions, PMVSDs were classified into perimembranous inlet (PMI), perimembranous outlet (PMO), and perimembranous trabecular (PMT) type [14]. Perimembranous confluent (PMC) type was defined as a large defect with extension to both inlet and outlet parts of the ventricular septum. The presence of ATVMS was graded as grade 0, absent; grade 1, tumor-like shape formed but not attached to the top of the tricuspid valve tissue; or grade 2, a tumor-like bulge shaped toward the right ventricle. Tricuspid regurgitation was described as mild, moderate, or severe, according to a previous study based on color-Doppler echocardiograph findings [15]. Some patients had associated complications during the follow-up, including left ventricular-to-right atrial shunt (LV-RA shunt), subaortic ridge with or without left ventricular outflow tract obstruction (LVOTO), aortic valve prolapse (AVP) with or without aortic regurgitation (AR) and hypertrophy of right ventricle outflow tract with or without right ventricular outflow tract obstruction (RVOTO). Indications for operation were 1) severe congestive heart failure (CHF) and echocardiography showing a PASP ≥60 mmHg; 2) chronic cardiac insufficiency for an extended time with poor feeding, tachypnea, poor weight gain, repeated pulmonary infection and continuous PH (36<PASP<60 mmHg) for 6 months; 3) chronic cardiac insufficiency without PH, where the defect size did not change significantly after at least 2 years' follow-up when patients have reached school age; 4) associated complications resulting in obstruction of the left or right ventricle, or moderate AR. Surgery was arranged when at least 1 of the indications was diagnosed.

### Follow-up

The outcome of interest was SC, defined as an absence of defect by 2-dimensional imaging and absence of flow across the ventricular septum, determined by pulsed or color Doppler echocardiography. Patients were followed up in 6-month or 1-year intervals until December 2009 (mean 20.9±15.0 months) in the derivation cohort and December 2012(mean 10.6±10.8 months) in the validation cohort, except in cases with just 1 echocardiogram, who were directly treated surgically because of severe CHF (Figure 1, Table 1).

**Table 1:** Number of patients with different follow up periods in derivation and validation cohort.

		n	Follow up period			
			≤1 year	1–3 years	3–5 years	>5 years
Derivation cohort	Spontaneous closure	343	143	152	39	9
	Persisting VSD	492	140	259	73	20
	VSD surgery	1038	716	271	43	8
Validation cohort	Spontaneous closure	34	24	10	/	/
	Persisting VSD	104	76	28	/	/
	VSD surgery	244	184	60	/	/

VSD, ventricular septal defect.

### Statistical analyses

All statistical analyses were performed using the SPSS software package (SPSS for Windows, Version 13.0. Chicago, SPSS Inc.). The main purpose of our analysis was to find demographic and echocardiographic variables that could be used to stratify PMVSD patients according to their probability of SC. A set of 11 baseline variables that were considered both medically relevant for SC prediction and easy to determine in usual clinical practice was identified. To extract the maximum information from our study population, we constructed our algorithm using the Cox proportional hazards model. This model has several advantages over other techniques, such as logistic regression: It accounts for variable duration of follow-up, censoring of subjects, proportionality of event occurrence, and time-to-event [16]. Our model can be used to generate estimates of SC probability of PMVSD over a specified time frame (e.g. 1 year and 3 years) and are useful for determining appropriate treatments for PMVSD patients. Univariate and multivariate analyses were performed by Cox regression. Parameters that were significant in the univariate analysis were entered in the multivariate analysis. Significant independent variables contributing to the prognosis of PMVSD patients were evaluated using stepwise selection methods. To increase the usefulness of the algorithm in clinical practice, the β-coefficient of each variable that independently predicted SC was standardized and rounded so a simple scoring system was developed. The general method for scoring scheme derivation is described in [17]. A receiver operating characteristic (ROC) analysis was performed to measure the discrimination of the prediction system. Patients were divided into three groups based on the distribution of their score values and SC probabilities. Survival curves of the three groups were constructed by Kaplan-Meier methods and analyzed with the log-rank test. The scoring system was then used to evaluate its validity for other patients with PMVSD, calculating the individual probability of each patient to have SC.

## Results

### Prognosis or survival analysis

Of the 1873 patients in the derivation group, 343 (18.4%) developed SC, whereas 1038 patients underwent surgery for their defect (including patch closure in 918 and device closure in 120 cases) and 492 patients with persisting VSD who had been observed for 2–82 months (mean 23.8±15.9 months, median 21 months) were managed conservatively (Table 1). From all 1038 surgically managed patients, 404 were severe CHF cases with only 1 echocardiographic examination (Figure 1). The overall cumulative defect remaining open rates were 91% at 1 year, and 79% at 3 years.

### Factors associated with spontaneous PMVSD closure

Table 2 and 3 report the characteristics of all patients and echocardiography data in the derivation cohort. First, we did a univariate analysis and found that initial contact age, defect diameter, ATVMS formation, diffuse shunt flow, associated complication and increased LVDD were significantly associated with prognosis in the derivation cohort. Next, input variables were evaluated by forward stepwise Cox regression analysis as being significant factors that contribute independently to the prognosis of PMVSD. All the input variables were considered as independent predictors for SC (Table 4).

**Table 2:** Baseline characteristics (categorical variables) of 1873 patients in the derivation cohort.

Categorical variables		n (%)
Sex	Male	1027 (54.8)
	Female	846 (45.2)
Additional abnormality	No	1034 (55.2)
	One	736 (39.3)
	More than one	103 (5.5)
Defect extension	PMI	317 (16.9)
	PMO	53 (2.8)
	PMT+I	993 (53.0)
	PMC	510 (27.2)
Shunt flow	Diffuse	1144 (61.1)
	Not diffuse	729 (38.9)
ATVMS formation	Grade 0	347 (18.5)
	Grade 1	890 (47.5)
	Grade 2	636 (34.0)
Tricuspid regurgitation	No	1453 (77.6)
	Mild	385 (20.6)
	More than mild	35 (1.9)
Associated complication	No	1300 (69.4)
	One	471 (25.27)
	More than one	102 (5.4)
Pulmonary hypertension	No	1421 (75.9)
	Yes	452 (24.1)
Increased LVDD	No	598 (31.9)
	Yes	1275 (68.1)

VSD, ventricular septal defect; ATVMS, aneurysmal tissue of the ventricular membranous septum; PMI, perimembranous inlet; PMO, perimembranous outlet; PMT+I, perimembranous trabecular and inlet; PMC, perimembranous confluent; LVDD, left ventricular end-diastolic dimension.

**Table 3:** Baseline characteristics (continuous variables) of 1873 patients in the derivation cohort.

Continuous variables	Mean±S.D
Initial contact age (months)	21.60±28.8
VSD diameter (cm)	0.77±0.25

**Table 4:** Results of multivariate Cox regression analysis in the derivation cohort.

	β-coefficients	Hazard ratio*	95% Confidence interval	P value
Initial contact age (months)	−0.054	0.947	0.939–0.955	<0.001
VSD diameter (cm)	−2.676	0.069	0.032–0.149	<0.001
Diffuse shunt flow	−0.413	0.661	0.513–0.852	<0.001
ATVMS formation	0.825	2.282	1.811–2.875	<0.001
Associated complication	−1.442	0.236	0.152–0.367	<0.001
Increased LVDD	−0.304	0.738	0.593–0.919	0.007

*Hazard ratio per unit increase.

VSD, ventricular septal defect; ATVMS, aneurysmal tissue of the ventricular membranous septum; LVDD, left ventricular end-diastolic dimension.

### Construction of Cox Model

The independent predictors, together with the β-coefficients of the Cox model, the hazard ratios, and the 95% confidence intervals, are shown in Table 4. Average 1-year defect remaining open rate was 0.989 and average 3-year defect remaining open rate was 0.948, which were estimated at the mean value of the variables. To estimate the probability of an individual with PMVSD occurring SC, the following formula was used:


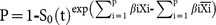
, with 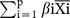 = −3.246 which can be done once using the regression coefficients and means of the variable. The SC probabilities of 1-year and 3-year estimate were formulated as followers: 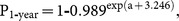


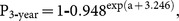
with a = ([−0.054× Initial contact age]−[2.676×VSD diameter]−[0.413× Diffuse shunt flow]+[0.825×ATVMS formation]−[1.442×Associated complication]−[0.304× Increased LVDD]).

For all binary variables, the presence of the variable is equivalent to ‘1’, whereas the absence of the variable is equivalent to ‘0’. For ATVMS formation, grade 0 is equivalent to ‘0’, grade 1 is equivalent to ‘1’, and grade 2 is equivalent to ‘2’. For associated complication, absent is equivalent to ‘0’, single is equivalent to ‘1’, and multiple is equivalent to ‘2’. For example, for a PMVSD patient with initial contact age of 6 months, VSD diameter of 0.3 cm, no associated complication (code 0), ATVMS formation of grade 2 (code 2), diffuse shunt flow (code 1), and increased LVDD (code 1), a is calculated as a = ([−0.054×6]−[2.676×0.3]−[0.413×1]+[0.825×2]−[1.442×0]−[0.304×1]) = −0.194, the 1-year probability of occurring SC is 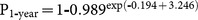 = 0.209, and the 3-year probability of occurring SC is 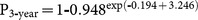 = 0.677.

### Development of a spontaneous closure predicting scoring system

**Figure 2:**
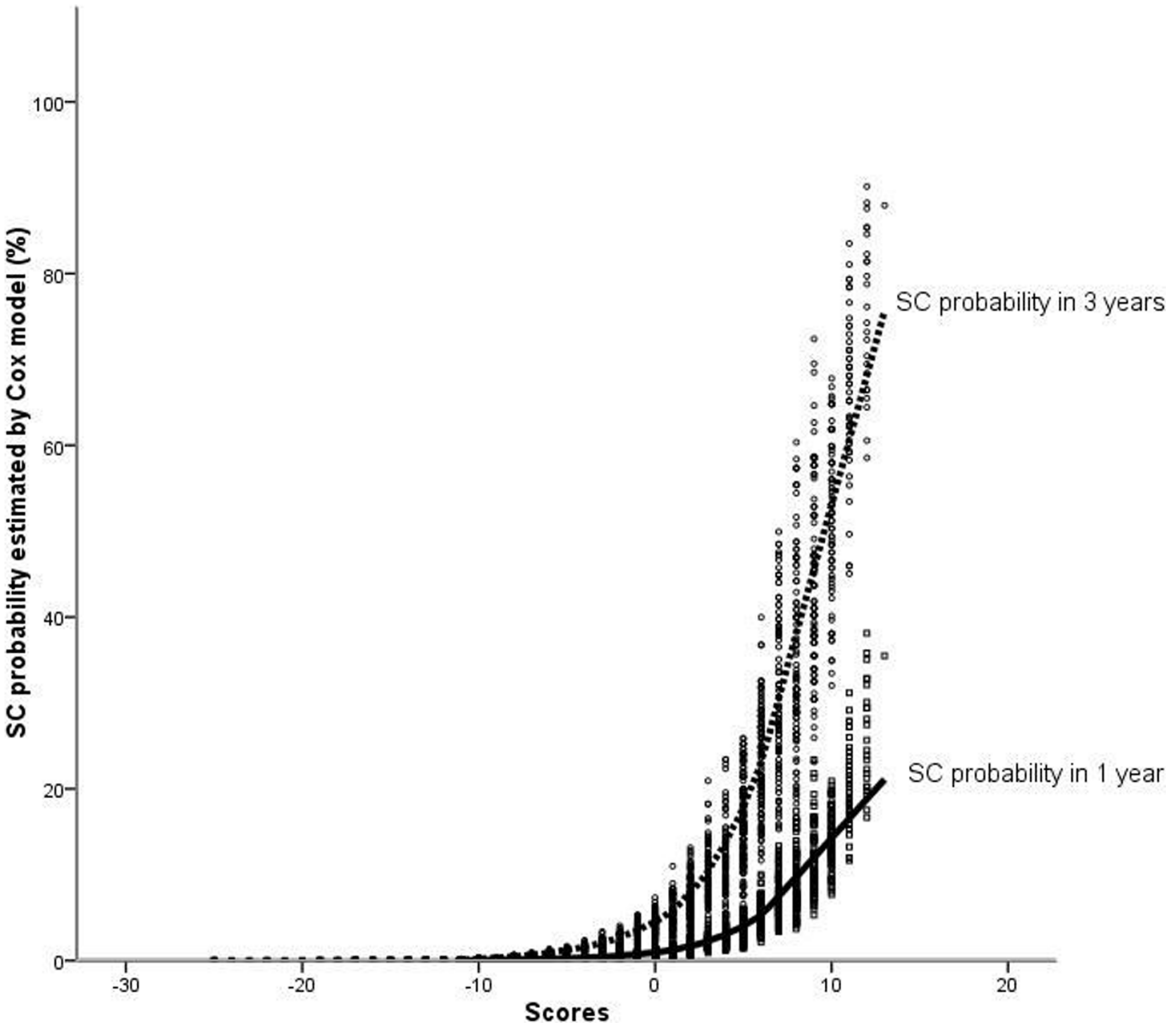
Plots show SC probabilities occurring within 1 year(□) and 3 years(○) among PMVSD patients in the derivative cohort, plotted against the scoring system. The LOESS fit lines (the solid line for 1-year and the dashed line for 3-year) using 50% fit plots show the trend of SC probability against the score. (SC, spontaneous closure).

**Figure 3:**
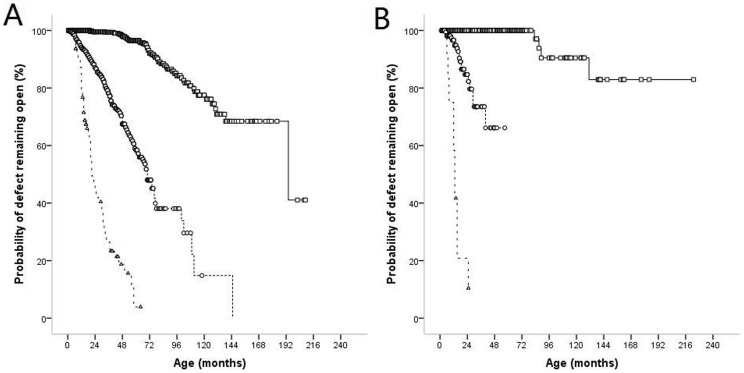
Comparison among PMVSD patients of high probability(△), intermediate probably(○) and low probability(□) occurring SC by Kaplan-Meier's method. A: Probability of defect remaining open in the deviation cohort (p<0.001). B: Probability of defect remaining open in the validation cohort (p<0.001).

With the derived data, we developed a scoring system for prediction of SC, and different score values for variables were established according to their regression coefficients with the method described in [17] (Table 5). Table 6 shows the 1-year and 3-year SC probability associated with each score. For the above case, the scoring system gives an estimated SC probability of 25.18% in 1 year, and 75.35% in 3 years, indicating that there is good agreement between the estimates produced by the Cox model and the scoring system. We plotted the SC probability in 1 year and 3 years as calculated with the Cox model for each patient against the score (Figure 2). As shown in Figure 2, higher scores resulted in increased probability of SC. Excluding all censored data (ie, individuals who did not complete the 3 years of follow-up without having SC), the area under the ROC curve obtained with the scoring system was 0.831 (95% CI 0.804–0.858, p<0.001), indicating that the scoring system could discriminate well between patients who had SC and those who did not. Finally, for comparison, all patients were divided into 3 groups based on SC probabilities against scores (Figure 2): (1) patients with a score ≤0 (low probability); (2) patients with a score between 1 and 10 (intermediate probability); (3) patients with a score>10 (high probability). A survival curve was constructed for each group. Figure 3a shows that the high-probability group had a lower probability of defect remaining open than the low-probability group (p<0.001, Figure 3A). It was proven that increased scores were associated with significantly increased SC probabilities. The observed rates of SC (and the expected probability in 3 years) in these strata were 6.5% (<4%) for low probability, 22.6% (4.82–53.35%) for intermediate probability, 75.9% (64.42–92.37%) for high probability (Table 7).

**Table 5:** Scoring system for prediction of spontaneous PMVSD closure.

Variables		Score
Initial contact age	≤1 y	4
	1–2 y	2
	2–3 y	0
	3–4 y	−2
	4–5 y	−4
	5–6 y	−6
	>6 y	−8
VSD diameter	≤0.30 cm	5
	0.31–0.60 cm	3
	0.61–0.90 cm	0
	0.91–1.20 cm	−3
	>1.20 cm	−5
Associated complication	No	0
	One	−5
	More than one	−10
Diffuse shunt flow	Yes	−1
	No	0
ATVMS formation	Grade 0	0
	Grade 1	3
	Grade 2	5
Increased LVDD	Yes	−1
	No	0

PMVSD, perimembranous ventricular septal defect; VSD, ventricular septal defect; ATVMS, aneurysmal tissue of the ventricular membranous septum; LVDD, left ventricular end-diastolic dimension.

**Table 6:** Probability of SC associated with each score.

Total Scores	1-year probability of SC (%)	3-year probability of SC (%)	Total Scores	1-year probability of SC (%)	3-year probability of SC (%)
−25∼−10	<0.05	<0.2	3	1.86	8.68
−9	0.05	0.24	4	2.52	11.58
−8	0.07	0.32	5	3.40	15.36
−7	0.09	0.43	6	4.57	20.23
−6	0.12	0.59	7	6.15	26.39
−5	0.17	0.79	8	8.24	33.98
−4	0.22	1.08	9	11.00	43.03
−3	0.30	1.45	10	14.61	53.35
−2	0.41	1.97	11	19.27	64.42
−1	0.56	2.66	12	25.18	75.35
0	0.75	3.58	13	32.51	85.02
1	1.02	4.82	14	41.31	92.37
2	1.38	6.48			

SC, spontaneous closure.

**Table 7:** Estimated probabilities and observed SC rates in derivation and validation cohort according to score groups.

Score group	Estimated probability in 1 year (%)	Estimated probability in 3 years (%)	Derivation cohort (n = 1873)*			Validation cohort (n = 382)*		
			Total	SC	Observed SC rate (%)	Total	SC	Observed SC rate (%)
Low probability (≤0)$	<1	<4	759	49	6.5	187	6	3.2
Intermediate probability (1–10)#	1.02–14.61	4.82–53.35	1035	234	22.6	183	20	10.9
High probability (>10)$	19.27–41.31	64.42–92.37	79	60	75.9	12	10	83.3

SC, spontaneous closure;

*p<0.001 indicates the difference of observed spontaneous closure rates among score groups both in the derivation and the validation cohort.

$ p>0.05 indicates the difference of observed spontaneous closure rates in low probability and high probability score groups between the derivation and validation cohorts.

# p<0.05 indicates the difference between the derivation and validation cohorts of observed spontaneous closure rates in intermediate probability patients.

### Validation of the spontaneous closure prediction scoring system in a new patient cohort

Of the 382 patients in the validation cohort, 34 patients (8.9%) had SC, which was much lower than in the derivation cohort (p<0.001). 244 patients underwent surgery for their defect and 104 patients had persisting VSD whose follow up period was much shorter than that in the derivation cohort (Figure 1, Table 1). The area under the ROC curve was 0.863 (95% CI 0.785–0.941, p<0.001), indicating a good prediction of the scoring system. In the validation cohort, 187 (49%) of the patients were classified as low probability cases, 183 (47.9%) as intermediate probability cases, and 12 (3.1%) as high probability cases. Similar to the data shown in Figure 3A, increased scores were associated with significantly increased SC probabilities (p<0.001, Figure 3B). The observed rates of SC (and the expected probability in 3 year) were 3.2% (<4%) for low probability, 10.9% (4.82–53.35%) for intermediate probability, 83.3% (64.42–92.37%) for high probability (Table 7). The observed rate of SC of the intermediate probability group in the validation cohort was much lower than that in the derivation cohort (10.9% vs. 22.6%, p<0.05), while other scores did not differ significantly between the 2 cohorts, indicating the reliability of the score grouping.

## Discussion

Surgery is considered the gold standard for the treatment of VSD, but it is associated with considerable morbidity and mortality [18]. Zhuang et al. reported a postoperative mortality rate of 5.65%, mainly due to pulmonary hypertensive crisis and arrhythmia [19], and Demirag et al. noted 6.4% early postoperative deaths [20]. Particularly, young children with low body weight were reported to be at high risk, with 14.29% and 14.71% mortality in patients aged less than 1 year and weighing less than 7 kg, respectively [21]. We suggest that surgical PMVSD correction should be reserved for cases in which SC cannot be expected. Additionally, in China, due to non-advanced medical technology in underdeveloped areas with non-specialized cardiovascular physicians, the transfer and accommodation of patients and their attending families into distant specialized hospitals are particular obstacles. Our simple scoring system, which includes all the factors that are routinely used for the same decision-making process might help to predict spontaneous VSD closure and indicate necessary operations.

According to our analysis, initial contact age, defect diameter, diffuse shunt flow, ATVMS formation, associated complication and increased LVDD yielded the best prediction model for SC of PMVSD. In the published literature, defect size has been considered as the major determinant of SC of isolated VSD [22]. Shirali et al. [6] developed a model in which the indexed VSD cross-sectional area indicated a high probability of SC (≤0.5 cm^2^/m^2^) or surgery (≥1.0 cm^2^/m^2^). Our findings are consistent with these studies, in that defect diameters ≤0.30 cm had a much higher probability of SC than larger ones.

ATVMS has been reported to be an important factor for SC or defect size diminution, with more favorable PMVSD prognosis [4], [23], [24], which is in accordance with our findings. However, in contrast to previous reports, we classified ATVMS formation into 3 grades in which no ATVMS formation (grade 0) and tumor-like bulge shaped toward the right ventricle (grade 2) were significantly associated with spontaneous PMVSD closure. Although Shirali et al. noted that the likelihood of SC could be more accurately predicted by the indexed defect size than ATVMS formation [6], our study suggests that both VSD diameter and ATVMS formation are significant predictors.

PMVSDs have been associated with serious complications, such as LV-RA shunt [4], [8], AVP [2], AR [2], subaortic ridge [2], RVOTO [25] and infective endocarditis [8] having a negative effect on the SC rate [2], [8], [25], which is in line with our findings.

Although the key elements in terms of SC were VSD diameter, ATVMS formation, and no associated complications, other factors, including initial contact age, diffuse shunt flow and increased LVDD also played roles. The significantly higher rate of SC in patients with initial contact age ≤1 year is consistent with most previous reports, which showed that the rate of SC is highest in the first year after birth. Diffuse shunt flow, which might reflect a hemodynamic change or a communication between the 2 ventricles, should also be considered as a factor associated with the outcome. Increased LVDD was a negative predictor for SC, which is consistent with previous studies suggesting that enlarging left ventricular size is an indication for surgical repair [26]. However, pulmonary hypertension did not appear in our prediction model and scoring system, indicating that PH is not a prognostic factor but rather a manifestation of the clinical course in patients with VSD.

Our system has good discriminatory ability, with an area under the ROC curve of 0.83 and it is possible to group patients according to their scores in high-probability, intermediate-probability and low-probability in both the derivation and the validation cohort. Patients predicted to have a low probability have the poorest prognosis and have to be managed surgically as early as possible to avoid severe CHF. Patients predicted to have an intermediate probability could be managed operatively with elective procedures and their parents could be informed appropriately; thereby allowing them to prepare mentally and financially. For patients who are predicted to have a high probability, close follow-up by a pediatrician is recommended, in order to reduce parental anxiety.

In the validation group, particularly for patients with intermediate probability, the observed SC rate was lower than estimated. First, the follow-up period was much shorter in the validation cohort, as the start time was January 2010. Second, with the rapid development of sophisticated techniques, more parents agreed to operations for small traumata with low risk and fewer post-operative complications, rather than to wait for a natural closure of the defect in their children. It is important to note the possibility of overcorrecting PMVSD by surgery or intervention, since a group of patients with intermediate probability of SC could avoid operative management. Although the scoring system at different times on the vertical comparison had certain differences, the SC rate of PMVSD was still higher, with higher scores during the same period in the horizontal comparison.

Limitations of our study include the fact that the defect size was measured by 2D echocardiography, resulting in lower accuracy, due to the circular VSD shape. In addition, the model does not include clinical information like X-ray, EKG variables, or the effects of medications such as diuretics, which may have an influence on SCs. Finally, the scoring system needs longer follow-up to validate its feasibility. Not all patients with high probability according to their score had a SC at the last visit, indicating that the scoring system just provides complementary clinical information, which might help the pediatrician to make predictions about their future status, but it cannot provide an exact prognosis. All information, including clinical manifestation, electrocardiogram, chest X-ray, and the scores discussed above should be considered together to predict the outcome of a patient with PMVSD. Close follow-up of all patients is also recommended.

## Conclusions

Age at initial contact, VSD diameter, diffuse shunt flow, ATVMS formation, increased LVDD and associated complications can be independent predictors of spontaneous PMVSD closure. Outcome can be estimated by our new scoring system, using these independent predictors, and such scoring of PMVSD patients may facilitate appropriate planning by pediatricians.
